# The underwhelming German life expectancy

**DOI:** 10.1007/s10654-023-00995-5

**Published:** 2023-04-25

**Authors:** Domantas Jasilionis, Alyson A. van Raalte, Sebastian Klüsener, Pavel Grigoriev

**Affiliations:** 1 Laboratory of Demographic Data, Max Planck Institute for Demographic Research, Konrad Zuse str. 1, Rostock, DE-18057 Germany; 2 Independent Research Group of Lifespan Inequalities, Max Planck Institute for Demographic Research, Konrad Zuse str. 1, Rostock, DE-18057 Germany; 3 Research Area of Demographic Change and Longevity, Federal Institute for Population Research (BIB), Friedrich- Ebert-Allee 4, Wiesbaden, DE-65185 Germany; 4 Research Group Mortality, Federal Institute for Population Research (BIB), Friedrich-Ebert-Allee 4, Wiesbaden, DE-65185 Germany

**Keywords:** Life expectancy, Causes of death, Germany, High-income countries, Divergence

## Abstract

This article contributes to the discussion on the determinants of diverging life expectancy in high-income countries, with a focus on Germany. To date, much of this discourse has centered around the social determinants of health, issues of healthcare equity, poverty and income inequality, and new epidemics of opioids and violence. Yet despite doing well on all of these metrics and having numerous advantages such as comparatively strong economic performance, generous social security, and an equitable and well-resourced health care system, Germany has been a long-time life expectancy laggard among the high-income countries. Using aggregated population-level mortality data for Germany and selected six high-income countries (Switzerland, France, Japan, Spain, the United Kingdom, and the United States) from the Human Mortality Database and WHO Mortality Database, we find that the German longevity shortfall is mainly explained by a longstanding disadvantage in survival among older adults and adults nearing statutory retirement age, which mainly stems from sustained excess cardiovascular disease mortality, even when compared to other laggard countries such as the US and the UK. Patchy contextual data suggests that the unfavorable pattern of cardiovascular mortality may be driven by underperforming primary care and disease prevention. More systematic and representative data on risk factors are needed to strengthen the evidence base on the determinants of the controversial and long-standing health gap between more successful countries and Germany. The German example calls for broader narratives of population health that embed the variety of epidemiological challenges populations face around the globe.

**Supplementary Information:**

The online version contains supplementary material available at 10.1007/s10654-023-00995-5.

## Introduction

Long and healthy lives are a key indicator of success or failure in advancements of human development [1, 2]. Stalls or declines in life expectancy in the UK and the USA in the years preceding the Covid-19 pandemic have accordingly struck a worrisome tone [3–5]. Perhaps because these two countries are liberal regimes characterized by comparatively weaker social safety nets [6], much of the narrative comparing health and life expectancy divergences across high income countries has focused around the role of social policies, poverty and inequality, and health care equity [7–12]. In addition, the US-based studies have demonstrated the important role played by an epidemic of opioid-overdose mortality [3, 4], while the UK-studies have focused on the impact of smoking-attributable mortality among females, excess mortality at working ages, and the overall negative impact of austerity policies on population health [5, 11].

It is important to put these findings into a broader context. Seemingly unnoticed in these high-profile studies of life expectancy stalls or reversals are countries that have failed to improve their low life expectancy rankings over time: i.e., the steady laggards, who do not neatly fit into the same narrative. As a result, there is strikingly little awareness that Germany is part of this group with underwhelming life expectancy. Existing comparative mortality research on Germany has mostly focused on the “success story” of life expectancy levels in eastern Germany having caught up to those in western Germany after reunification in 1990 [13–15].

In 2019, Germany ranked 14 out of 15 in male life expectancy (ahead of Portugal) and 13 out of 15 in female life expectancy (ahead of the UK and Denmark) out of the group of EU-15 countries (EU members before 2004, including the UK) [16]. More broadly, German men experienced a near 3-year life expectancy gap to the worldwide highest-ranked country Switzerland, and German women a 4-year gap to the highest ranked Japan [16]. These life expectancy shortfalls have hardly shifted since reunification in 1990, when they were around 3.5 years for both sexes. Furthermore, the German life expectancy disadvantage has a relatively longstanding history, which has been in existence at least since the mid-20th Century (the earliest period for which constant mortality data series are available). (Supplementary Fig. S1; Supplementary Table S1).

Germany’s long-term shortfall in life expectancy relative to the longevity leaders [16] is striking when weighed against Germany’s many assets, including its outstanding macroeconomic performance; i.e., its stable economic growth, low poverty and unemployment, and low governmental debt [17]. It is widely acknowledged that the German health care system is both equitable and highly developed in terms of resources and technology [18–20]. Germany also has among the highest levels of per capita expenditures on health care worldwide [17]. Nevertheless, health care, together with its specific characteristics (including financing), is only one of the components that influence the longevity differences between and within countries [21]. There are other factors that may contribute to the cross-country differences in longevity, such as differences in educational levels, the distribution of wealth (income), social cohesion, the effectiveness of the social welfare system; as well as the history of lifestyle-related behaviors, such as smoking, alcohol consumption, diet, and physical exercise [10, 14, 22, 23].

In this study we investigate the longstanding German disadvantage in life expectancy with respect to six high-income countries using demographic methods and cause-of-death analysis. We selected six high-income countries to compare to Germany for the following reasons: (1) Switzerland, because it has strong linguistic and cultural ties with Germany, and is a consistent leader in life expectancy for men; (2) France, as a neighboring country that is known to have particularly low mortality at older ages [16]; (3) Japan, because it has reported the highest life expectancy in the world for women for several decades; (4) Spain, as a country which has made rapid progress in the life expectancy rankings, and is forecasted to overtake Japan as the next life expectancy leader [24]; and, finally, (5) the United Kingdom and (6) the United States, because they have recently experienced well-documented mortality slowdowns [3–5]. These results are complemented by a discussion on the potential determinants of the observed patterns, pulling together the most comparable contextual evidence. We aim to ascertain whether similar determinants of higher mortality are at play in Germany compared to established life expectancy laggards such as the US and the UK, or whether Germany has forged its own underwhelming path of life expectancy progress.

## Methods

### Data source

Life expectancy estimates used for comparative analyses stem from life tables obtained from the Human Mortality Database (HMD) [16]. The HMD was also the data source for all-cause death and population exposure-to-risk counts used to derive age-specific death rates for Germany and comparator countries for the years 1990 to 2020. The advantage of using the HMD data is related to (a) the uniform methods used to harmonize and adjust input data and (b) the application of the same life table construction methodology [25]. It was particularly important to ensure a rigorous source of mortality data for Germany. Prior the Population Census 2011, German mortality data were affected by the systematic overestimation of population denominators due to misreporting of migration events [26]. The last census revealed an overestimation of the population size by 1.5 million individuals. Because the Federal Statistical Office did not produce adjusted intercensal population estimates accounting for this inconsistence, the HMD team carried out a special project on data harmonization [27]. Our study benefits from the harmonized mortality series produced for Germany within this project.

We chose 1990 as a base year because it was the first year in which Germany was unified. The age- and cause-of-death data were obtained from the WHO Mortality Database [28] for the period 1990–2016, for which cause-specific data were available for all countries. To ensure better data comparability, we restricted our analyses only to seven major groups of causes of death (cardiovascular system diseases (ICD9: 390–459, ICD10: I00-I99), neoplasms (ICD9: 140–239, ICD10: C00-D48), external causes of death (ICD9: 800–999, ICD10: V01-Y89), respiratory system diseases (ICD9: 460–519, ICD10: J00-J98), digestive system diseases (ICD9: 520–579, ICD10: K00-K92), infectious diseases (ICD9: 001-139, ICD10: A00-B99), all other (remaining) causes of death. Age-standardized death rates were estimated for these groups of causes of death, with the 1976 European Population Standard used as the standard. The data on health expenditures per capita, hospital utilization for CVD conditions and related risk factors, and health behavior and risk factors were obtained from the OECD database [17, 20].

### Statistical analysis

We used these data to construct abridged period life tables using standard life table methods [29]. Period life table functions, including the remaining life expectancy after a certain age, refer to the current mortality conditions under the assumption that these conditions will remain fixed throughout the entire life of a hypothetical cohort (N = 100,000) [30]. Standard demographic methods [31] of age decomposition of life expectancy difference were used to obtain exact contributions of differences in death rates within each age interval to the total gap in life expectancy at birth between Germany and each comparator country. The negative values of the estimated age-specific contribution (in years of life expectancy difference) indicates that Germany has a higher mortality than in a comparator country, whereas the corresponding positive contributions indicate a lower mortality in Germany. This analysis was complemented by age and cause decomposition, further disentangling the age-specific contributions into the contributions of differences in cause-specific mortality [31]. The total sum of age- and cause-specific components refers to the total contribution of the differences by each group of causes of death to the total gap in life expectancy at birth. The dispersion in ages at death was measured using the lifespan disparity (e†) measure. Lifespan disparity is interpreted as the average years of life lost at the time of death due to a premature death —when ages at death are highly spread out across individuals (i.e. towards younger ages), the average years of life lost at death is higher [32]. Measures of dispersion are especially useful in uncovering mortality crises, particularly among younger adults. All calculations and analyses were performed using R software (version 4.0.2).

## Results

### Life expectancy and national health care expenditures

**Fig. 1:**
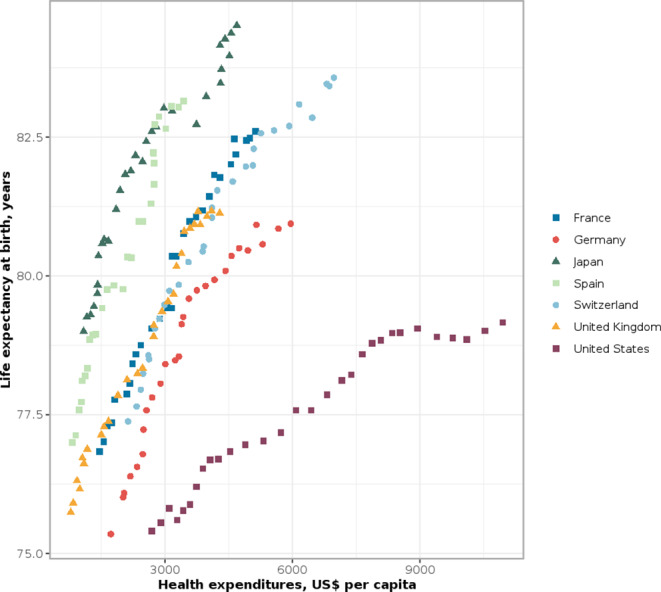
Health expenditures per capita (in USD) and life expectancy at birth in Germany and selected high-income countries, 1990–2019

Figure 1 illustrates relationships and discrepancies between health care expenditures per capita and life expectancy outcomes in Germany and selected high-income countries. The estimates suggest that maintaining higher health expenditures in the USA and Germany have not led to any longevity advantages against countries with smaller scale health financing. On the contrary, in terms of life expectancy, the USA and Germany can be classified in the worst positions (Fig. 1). A different pathway can, for example, be observed in Germany’s neighboring country Switzerland, where similar systematic increases in health expenditures were accompanied by a convergence of the country’s life expectancy with that of the countries with the highest longevity. Meanwhile, the leading longevity countries (Japan and Spain) manage to sustain the highest life expectancy levels at substantially lower health expenditures. Even the UK, being another longevity laggard in the selected group of countries, shows a slight life expectancy advantage against Germany despite notably lower health expenditures (Fig. 1).

More insights about longevity divergences between Germany and the other six high-income countries can be drawn from inspection of annual changes in sex-specific life expectancy at birth and at age 65 between 1990 and 2020. Among these countries, Germany had the lowest life expectancy at birth for both males and females in 1990, and remained among the lowest-performing countries until the onset of the Covid-19 pandemic in early 2020. During this period, Germany managed to overtake only the US (both sexes) and the UK (females only).

**Fig. 2:**
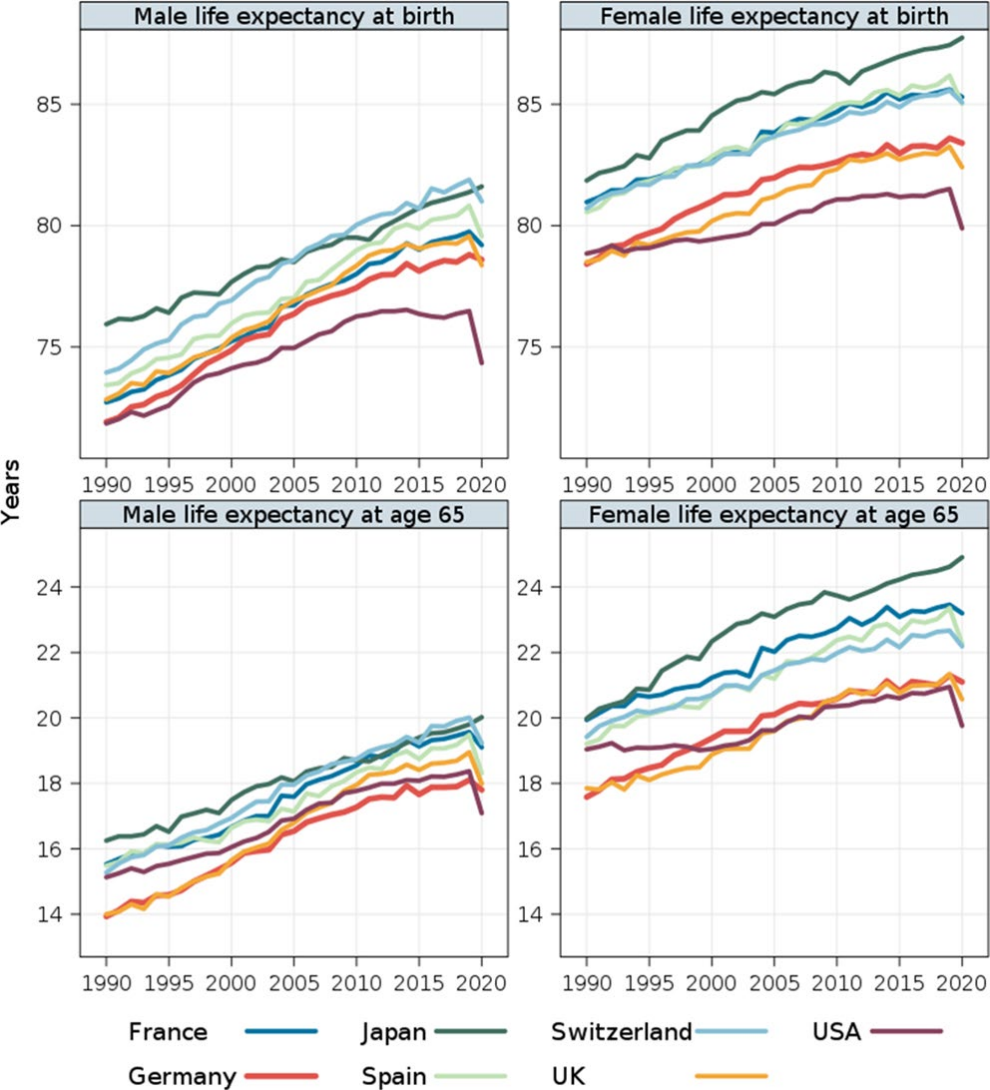
Trends in life expectancy at birth and at age 65 in Germany and other six selected high-income countries, 1990–2020

The poor performance of Germany is even more visible when we look at life expectancy at age 65. Among the selected high-income countries, Germany has reported the lowest life expectancy at age 65 for males since the mid-2000s. For German females, initial rapid improvements in life expectancy at age 65 decelerated in the second half of the 2000s. As a result, the life expectancy of females in Germany has converged to that of females in the laggard group consisting of the US and the UK. The most recent slowdown in life expectancy improvements in Germany has also contributed to a further divergence of German life expectancy from that of the longevity leaders, including Japan, Switzerland, Spain, and France (females only). For example, between 2006 and 2019, the life expectancy gap between Germany and Spain more than doubled for males (from 1.0 to 2.1 years), and increased from two years in 2006 to three years in 2019 for females. Finally, it is worth noticing that despite overall poorer longevity performance, between 2019 and the first pandemic year 2020, Germany experienced much less pronounced life expectancy declines than those observed in Spain, the UK, and even Switzerland (Fig. 2).

### Age decomposition of life expectancy

**Fig. 3:**
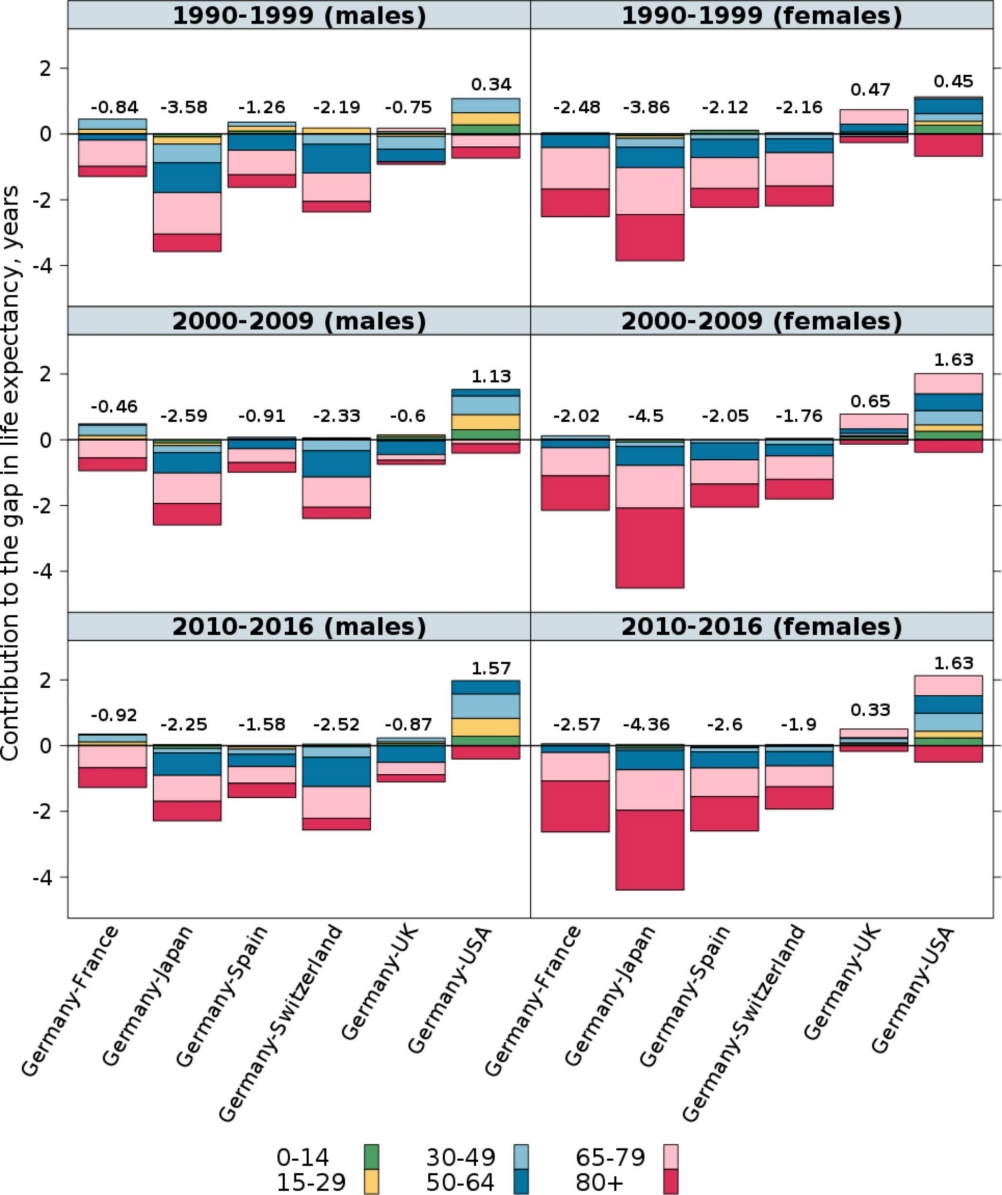
Age-specific contributions to the total difference in life expectancy at birth (values above the bars) between Germany and each of the other selected high-income countries, 1990-99–2010-16

Figure 3 compares life expectancy at birth in Germany with that in the six selected countries using the three aggregated periods of 1990–1999, 2000–2009, and 2010–2016. These estimates show that between 1990–1999 and 2010–2016, the life expectancy gap between Germany and the four leading countries increased overall, despite some narrowing during the 2000s. However, the longevity differences between Germany and these four countries have been smaller for men than for women (except Switzerland). Figure 3 highlights the patterns of the age-specific contributions of mortality differences to the total gap in life expectancy at birth between Germany and the remaining six countries. Generally, this evidence points to the importance of increased mortality in Germany at ages 65 and older.

Over the study period, higher mortality at ages 65–79 among men was consistently the biggest contributor to the life expectancy gap between Germany and Japan, Switzerland, Spain, France, and the UK; while the second-largest contributor to this gap was excess mortality among men at ages 50–64 (except a comparison to France). The pattern was different for women. Among women, higher mortality at ages 65–79 also contributed considerably to the life expectancy gap. However, unlike for men, for women the contributions of higher mortality at ages 80 + were decisive in explaining the longevity gap between Germany and the four leading countries, particularly in the most recent period. The decomposition results also revealed differences in the age pattern of mortality between Germany and the UK and the US: e.g., Germany had lower mortality over most age groups, but higher mortality at ages 80 and older (Fig. 3).

**Fig. 4:**
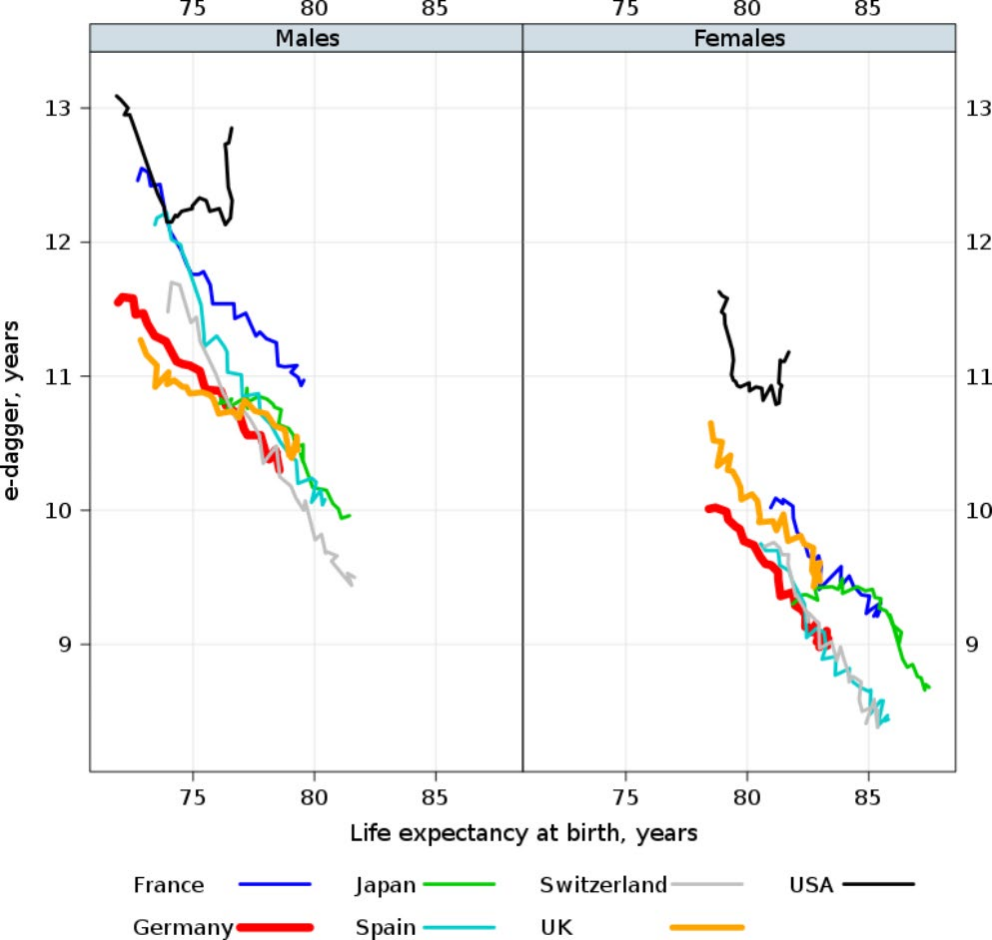
Changes in life expectancy at birth and lifespan disparity (e-dagger) in Germany and other high-income countries, 1990–2019

The dispersion of ages at death is lower in Germany than it is in the other life expectancy laggards, such as the UK and the US (Fig. 4). This dispersion, which is also known as lifespan inequality, is an important metric of both population heterogeneity and individual uncertainty in the timing of death. Figure 4 shows that the survival ages in Germany were becoming more equal over time, as life expectancy increased. This pattern is not seen in the US, where sharp increases in lifespan inequality have occurred in the last decade, due to midlife mortality increases accompanied by continued declines in mortality at older ages. Our results confirm (a) that the slower progress in life expectancy improvements in Germany cannot be explained by increasing heterogeneity in population health attributable to premature deaths at working ages; and (b) that it is mainly attributable to the mortality disadvantage among people at older ages, especially among those close to the modal (most typical) age at death.

### Continuing disadvantage in life expectancy: is it mainly attributable to cardiovascular diseases?

**Fig. 5:**
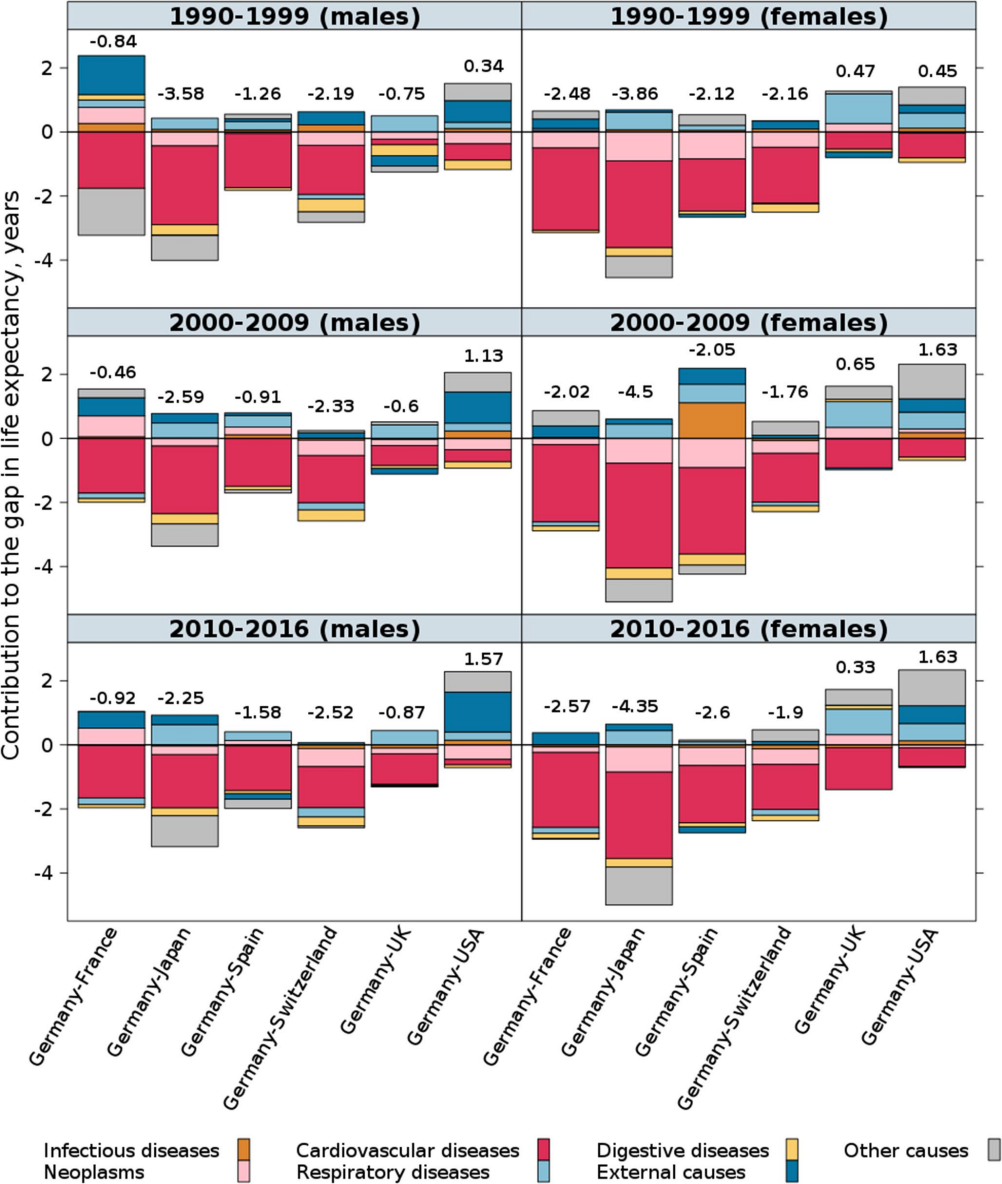
Cause-specific contributions to the total difference in life expectancy at birth (values above the bars) between Germany and each of the other selected high-income countries, 1990-99–2010-16

Because the overall progress in life expectancy at birth increasingly depends on mortality reductions in the most advanced age groups, we explore the assumption that the longstanding poor performance of Germany is attributable to Germany being less successful than other countries in treating chronic and aging-related diseases. Indeed, a cause-specific cause of death decomposition of the life expectancy disadvantage of Germany relative to the best-performing countries, including Japan, Switzerland, and Spain, shows that diseases of the cardiovascular system (CVD) have been the main contributors to this disadvantage (Fig. 5). This large cause-of-death group is found to be the most prominent in all of the study periods, despite the overall progress made in Germany in reducing CVD mortality. Over the study period, the share of the life expectancy shortfall that could be explained by excess CVD mortality in Germany remained high, or even increased further. A comparison of life expectancy at birth in Germany and the US is especially interesting. The higher male life expectancy in Germany was mostly attributable to lower adult mortality due to external causes of death, whereas the higher female life expectancy in Germany was largely explained by lower mortality from other (residual) causes of death, as well as lower mortality from respiratory diseases and mortality from external causes of death.

The growing life expectancy disadvantage of Germany relative to the best-performing countries is also evident when we look at the recent annual trends in standardized death rates due to cardiovascular diseases at ages 50–64 and 65+ (Supplementary Fig. S2). Germany started the 21st century with higher levels of CVD than the leading countries, and experienced slower progress or even signs of stagnation in the decline in CVD mortality after 2010. A particularly worrying trend over the study period was the systematically higher premature CVD mortality at ages 50–64 in Germany, with German males and females experiencing mortality that was twice as high that of their counterparts in the leading group of countries, excluding the US and the UK.

## Discussion

Despite its high-performing economy, equitable and cost-intensive advanced health care system, and well-developed social security system, Germany continues to be one of the worst performers among high-income countries in terms of life expectancy at birth. Germany’s performance with respect to life expectancy at age 65 is even worse, as on this indicator for males, Germany lags even behind the US, a well-known life expectancy underperformer in this group of countries. In terms of longevity, Germany and the US are substantially underperforming, even though their health expenditures are similar to or are much higher than those of many countries that belong to the longevity vanguard. The observed discrepancies between health care investments and population health outcomes are a warning sign for the sustainability of the entire health care system in Germany, as health care demand is likely to further increase in the near future due to population aging.

The study relies on harmonized and internationally comparable data allowing us to obtain reliable estimates of the age–specific mortality contributions to life expectancy gaps across countries. While all-cause mortality is highly comparable, cause-specific mortality data has its limitations. First, groupings of causes of death can be not directly comparable due to differences in coding practices. Second, the problems of temporal consistency may occur due to the changes in international classifications of causes of death (ICDs) [33]. To limit these impacts, we focused only on large groups of causes of death, for which these issues are more of a within- rather than between-category problem. Finally, we acknowledge that our insights on the potential determinants of the German life expectancy disadvantage are based largely on aggregated survey-based and routinely collected statistical data on socio-economic, health care, and health behavior characteristics. Although these measures may indicate some statistical associations, they cannot be used for making causal claims. Representative and internationally comparable data that provide consistent evidence on levels of and changes in major risk factors of chronic diseases in Germany are scarce.

The decomposition analyses applied in this study allowed us to identify the ages and the large groups of causes of death that are responsible for Germany’s longevity disadvantage relative to more successful high-income countries. The results suggested that Germany’s life expectancy shortfall is almost exclusively attributable to higher mortality among older adults and people of higher working ages, when chronic diseases become increasingly important. In particular, the findings showed that Germany is performing much worse in terms of cardiovascular mortality than other laggard countries, such as the US and the UK. Although this disadvantage can be partially explained by evidence indicating that Germany had a much higher initial level of cardiovascular mortality, its persistence is a worrying sign for hopes that Germany will experience further longevity improvements, and that its life expectancy levels will converge with those of other high-income countries in the near future.

In line with other prior studies, our study raises the question of why there are substantial problems in the early detection and prevention of cardiovascular system diseases in Germany, even though the country has a universally accessible and modern tertiary health care system [34, 35]. On one hand, Germany had the highest rankings in terms of health care resources (a large and well-developed hospital and rehabilitation network), technological supplies (MRTs), and public expenditures on curative medicine (supplementary Table S2). On the other hand, this country was ahead of the other countries in rates of hospitalization and of CVD treatment procedures, including transluminal coronary angioplasties and coronary artery bypass grafts (Supplementary table S2) [17]. From these statistics alone, we cannot determine whether this higher spending indicates that patients were being more actively treated in Germany than in other countries, or whether it was in response to a greater need for acute medical care. However, in what follows we argue that this excess in hospitalization and complex treatments may be attributable to a greater focus on curative medicine, rather than on effective prevention. Additional epidemiological evidence of the weak state of prevention efforts in Germany shows that the vast majority of patients with cardiovascular conditions were diagnosed too late and had other serious conditions. For example, about 80% of patients with heart failure had related comorbidities requiring complicated treatments with a high risk of poor outcomes prior to the event [34, 35]. These problems have been attributed to a weak focus on prevention and limited access to diagnostic tools, such as echo-cardiography [35]. Other studies have also found that in Germany, there is substantial room for improving prevention at the primary care level, which may lead to fewer hospitalizations and better health outcomes [36, 37].

The extent to which differences in behavioural risk factors exacerbate disparities in cardiovascular disease mortality between Germany and other countries remains uncertain. It has been suggested that many behavioral determinants associated with CVD, such as smoking and obesity, are patterned by year of birth (cohort) [38, 39]. Among German men, the most prolific smokers were those born in the 1950s; i.e., those who are now entering peak ages for cardiovascular mortality [40, 41]. A study based on the Socioeconomic Panel (SOEP) data reports that despite overall decreases in smoking prevalence, further increases were observed in smoking intensity among men born in 1950–1969 [42]. This particular cohort effect reported in this study was largely driven by men with lower socioeconomic status and by those residing in western Germany. Nevertheless, standardized death rates for lung cancer in Germany remained below the European average for men, and were only slightly above the average for women [43]. Thus, smoking in Germany is unlikely to be having an outsized impact on CVD mortality currently, although it might be expected to do so in the future. This is especially the case for eastern German women, who experienced a later smoking epidemic [40], or among the lower educated cohorts of men described above.

The evidence for the impact of other health behaviours on mortality is weaker. There are pockets of internationally comparable data indicating that people in Germany have relatively poor nutritional habits, including a low consumption of fruit and vegetables and a high consumption of sugar and sweeteners (supplementary Table S2). There is some evidence that public health policies in Germany are far less aggressive at targeting hazardous behavior linked to poor health outcomes. For example, during the 2000 and 2010s, Germany had one of the lowest international rankings among high-income countries in terms of public health policies, especially in the areas of tobacco, alcohol control, and nutrition [44–46]. There is also some evidence that the high levels of per capita alcohol consumption in Germany have resulted in higher alcohol-related mortality [47]. Unfortunately, most of these estimates do not rely on nationally representative data, and whether they are fully comparable across countries, is challenging to ascertain. Therefore, we tentatively conclude that there is no reliable evidence that traditional CVD factors such as smoking or overweight make a major contribution to the excess CVD mortality in Germany.

Seemingly, the unfavorable combination of weak preventive care policies and low rates of early detection of CVD is responsible for Germany having much higher hospitalization and mortality rates than many other high-income countries. International comparisons have suggested that German tertiary health care hospitals are able to provide adequate treatment, as they are comparatively well-resourced, and have advanced medical equipment and highly qualified personnel (supplementary Table S2). However, it is possible that many cardiovascular and diabetes patients are simply being treated too late, when their conditions have reached an advanced stage, and they are suffering from multiple serious comorbidities [34, 35].

There may be other hard to identify factors, such as cohort effects related to unfavorable early life conditions around and following WW2 [48, 49], driving excess German mortality. A proper analyses of comparative cohort vulnerability requires data based on extinct or almost extinct birth cohorts, for which there is no reliable German data. Instead, we decomposed gaps in temporary (partial) life expectancy between the exact ages 40 and 90 across countries into their age-cohort contributions. This decomposition of the differences in cohort temporary life expectancies between Germany and comparator countries did not reveal any clear, specific cohort patterns (Supplementary Figure S3). However, it should be understood that these data do not cover potentially vulnerable (or selective and robust) cohorts born around or before WW2. It should also be noted that East and West Germany had to be combined into a hypothetical unified Germany for the years these cohorts lived prior to 1990, which makes us hesitant to put strong interpretations on these results. Other evidence, based on heterogeneous data from earlier periods and strong assumptions, suggests that there was a strong decrease in life expectancy at birth for cohorts born around 1943–1947 [48]. Yet the study highlights that this decrease is solely attributable to the peaking infant mortality, whereas any significant decline in life expectancy was not observed for the same cohorts at ages 1 and 65. Moreover, if high CVD mortality was mostly a function of poor early-life conditions, then we would expect countries that suffered severe domestic hardship during WW2 to systematically have a higher share of CVD mortality than those less affected, which we have shown is not the case.

Life expectancy at the country level also depends on the magnitude of the mortality differences across regions and population groups. For example, the extent to which persisting east-west socioeconomic disparities impact the national health outcomes remains a focal point of discussions in Germany [13–15]. Eastern Germany is still affected by specific health problems, including a pronounced level of excess mortality at adult working ages among men [50]. On the other hand, in eastern Germany, the life expectancy disadvantage against the western part has been substantially reduced for males (1.2 years in 2019), whereas eastern German females even gained a slight advantage (0.1 years) against their western counterparts [16, 50]. This east-west convergence in longevity has coincided with Germany reporting low overall levels of regional inequality in longevity compared to other high-income countries [51–54].

Given the ongoing increases in health care costs, the long-standing and potentially growing health disadvantage of Germany relative to the countries with the highest longevity should be of great concern for scientists, politicians, and health care stakeholders. It is also important to stress that whether Germany experiences further systematic longevity advances, and catches up to the countries with higher life expectancy, will increasingly depend on (a) the country’s success in reducing mortality at more advanced ages (i.e., beyond ages 80 and 90), and in (b) combatting aging-related diseases, such as Alzheimer’s disease [21, 55]. While Germany is entering this new phase of epidemiological development, it is still dealing with the ongoing burden of excess mortality at older working ages related to the lack of progress in reducing premature cardiovascular mortality. The persistent burden of cardiovascular system diseases and the emerging need for further progress in fighting aging-related diseases will create even greater sustainability challenges and threats to an increasingly costly health care system. There are other worrying signs of potential obstacles to sustainable social and health development in Germany. In particular, income inequality has been rising, and health problems are increasingly concentrated in the lower socioeconomic groups at adult and older ages [17, 56, 57].

The high life expectancy levels in the vanguard countries, such as Japan and Switzerland, show that there is a substantial room for Germany to further improve the health of its population. Given its advantages in terms of its economic progress and health care infrastructure and financing, Germany could do much better. Although the well-resourced German health care system may ensure better capacity in times of unexpected and massive health challenges such as the Covid-19 pandemic, making more sustainable progress on health, and ensuring that life expectancy in Germany converges with that of the countries with the highest longevity levels, would require substantial additional efforts. These future changes will evolve in the context of rapid population aging and its consequences for society and the health care sector. It is evident that the primary areas of focus for Germany should include tackling the very high burden of premature morbidity and mortality due to cardiovascular system diseases. However, in order to achieve the progress in the area, more population-level evidence and more in-depth research are needed to understand and address this long-standing public health challenge in Germany.

This study has highlighted that having a comparatively equitable and well-resourced health care system, coupled with the lowest level of income inequality and second-lowest level of poverty among G7 countries has not resulted in above-average health outcomes at the population level. This calls for broader narratives of population health that embed the variety of epidemiological challenges populations face around the globe.

## Electronic supplementary material

Below is the link to the electronic supplementary material.

Supplementary Material 1

Supplementary Material 2

Supplementary Material 3
