# Supplementary material for: The underwhelming German life expectancy
Source: Eur J Epidemiol. 2023 Apr 25;38(8):839–50. doi: 10.1007/s10654-023-00995-5 (PMC10129301; doi:10.1007/s10654-023-00995-5)
Supplement: Supplementary file 1 — Supplementary Material 1 [file 10654_2023_995_MOESM1_ESM.pdf]

Article: The underwhelming German life expectancy. European Journal of Epidemiology. Authors: Domantas Jasilionis, Alyson van Raalte, Sebastian Klüsener, Pavel Grigoriev.
The corresponding author: Domantas Jasilionis, Laboratory of Demographic Data, Max Planck Institute for Demographic Research, Rostock, Germany. E-mail: jasilionis@demogr.mpg.de

Supplementary table S2 Major characteristics of health system financing, resources, and performance and health risk factors in Germany (DEU), France (FRA), the United Kingdom (UK), Spain (ESP), Switzerland (SWI), the USA, and Japan (JPN). OECD estimates [17, 20] for 2005 and 2019 (or nearest year).

**Indicator**	**2005**							**2019**						
	**DEU**	**FRA**	**UK**	**ESP**	**SWI**	**USA**	**JPN**	**DEU**	**FRA**	**UK**	**ESP**	**SWI**	**USA**	**JPN**
**Health care financing**														
Expenditure on health, % of gross domestic product	**10.3**	10.2	8.4	7.7	10.0	14.6	7.8	**11.7**	11.1	10.2	9.1[p]	11.3	16.8	11.0[e]
Expenditure on health, per capita, in USD (PPPs)	**3430**	3265	2736	2212	4106	6443	2471	**6518**	5274	4500	3600[p]	7138	10949	4691[e]
Expenditure on pharmaceuticals / other medical non-durables, per capita, in USD (current PPPs)	**..**	..	..	..	..	..	..	**935**	589	461	505	794	1376	803
Out-of-pocket expenditure, % of current expenditure on health	**14.0**	7.4	13.3	21.8	27.1	13.9	15.7	**12.7**	9.3	15.9	21.8[p]	25.3	11.3	13.0[18]
Out-of-pocket expenditure, per capita, in USD (current PPPs)	**479**	241	364	482	1115	897	387	**828**	488	714	785[p]	1805	1238	592[18]
**Health care resources (personal, capacity, medical technology)**														
Practicing physicians (providing care directly) (per 1000 population).	**3.4**	3.1[e]	2.4	3.6	3.7[e]	2.4	..	**4.4**	3.2	3.0	4.4	4.4	2.6	2.5[18]
Total hospital beds (per 1000 population)	**8.5**	7.2	3.7	3.3	5.5	3.2	14.1	**7.9**	5.8	2.5[p]	3.0[p]	4.6	2.8[18]	12.8
Curative (acute) care beds (per 1000 population)	**6.4**	3.7		2.7	4.7	2.8	8.3	**6.0**	3.0		2.5[p]	3.6	2.5	7.7
Magnetic Resonance Imaging units (per million population)	**19.9**	4.8	5.4	..	..	26.6[06]	40.1	**34.5[18]**	15.4	..	17.6	..	40.4	55.2[17]
Computed Tomography scanners (per million population)	**29.5**	10.0	7.5	..	31.4[e,07]	34.0[06]	..	**35.3[18]**	18.2	..	19.2[p]	38.7	44.9	111.5[17]
**Pharmaceutical consumption for CVD conditions**														
Antiarrhythmics, Class I and III (daily dosage per 1000 inh. per day)	**2.3[d]**	10.9	2.1[de]	3.0[d]	..	..	..	**3.0[d]**	..	1.3[d,17]	3.2	..	..	..
Antihypertensives (daily dosage per 1 000 inhabitants per day)	**12.3[d]**	14.0	14.5[de]	8.6[d]	..	..	..	**14.0[d]**	..	13.6[d,17]	9.1	..	..	..
Beta blocking agents (daily dosage per 1 000 inhabitants per day)	**73.3[d]**	55.1	36.5[de]	18.6[d]	..	..	..	**82.2[d]**	..	29.2[d,17]	24.3	..	..	..
Lipid modifying agents (daily dosage per 1 000 inhabitants per day)	**36.8[d]**	82.2	100.1[d,07]	74.5[d]	..	..	..	**103.7[d]**	..	149[d,17]	125.1	..	..	..
**Surgical treatments for CVD conditions and related risk factors**														
Transluminal coronary angioplasty (total procedures per 100000 inhabitants)	**279.3**	180.5	98.6[e]	91.7	177.4	..	..	**419.5**	288.7	124.6	128.6	311	..	..
Coronary artery bypass graft (total procedures per 100000 inhabitants)	**79.9**	30.3	37.7[e]	18.1	47.3	..	..	**54.6**	28.4	22.2	16.4	39.3	..	..

Notes: [d] - different methodology or break in series; [e] - OECD estimate; [p] - provisional figure; [06] - 2006; [07] – 2007; [09] – 2009; [14] – 2014; [15] - 2015; [16] – 2016; [17] – 2017; [18] – 2018.

Supplementary table S2 (continued). Major characteristics of health system financing, resources, and performance and health risk factors in Germany (DEU), France (FRA), the United Kingdom (UK), Spain (ESP), Switzerland (SWI), the USA, and Japan (JPN). OECD estimates [17, 20] for 2005 and 2019 (or nearest year).

	2005							2019						
**Indicator**	**DEU**	**FRA**	**UK**	**ESP**	**SWI**	**USA**	**JPN**	**DEU**	**FRA**	**UK**	**ESP**	**SWI**	**USA**	**JPN**
**Hospital utilization for CVD conditions and related risk factors**														
Age-sex standardized hospitalization rate (per 100 000 population) for congestive heart failure	**347.0**	..	124.1[06]	146.7	299.2[06]	..	147	**393.8**	266.3[15]	107.8	157.1	402.9	411.7[18]	..
Age-sex standardized hospitalization rate (per 100 000 population) for hypertension	**229.1**	..	12.1[06]	9.2	102.4[06]	..	58.2	**254.5**	33.0	18	5.6	49.4	57.5[18]	..
Age-sex standardized hospitalization rate (per 100 000 population) for diabetes	**246.4**	..	..	66.8	149.5[06]	..	242.2	**206.1**	150.6[15]	80.9	50.4	106.9	226.0[18]	..
Male age-standardized rate (per 100 000 population) for diabetes lower extremity amputation	**19.8**	..	..	11.8	..	..	..	**12.5**	7.1[15]	4.8[17]	11.8	5.0[15]	47.1[18]	..
Female age-standardized rate (per 100 000 population) for diabetes lower extremity amputation	**9.9**	..	..	5.1	..	..	..	**4.0**	2.1[15]	1.5[17]	3.1	1.5[15]	16.7[18]	..
Hospital discharges for diabetes mellitus (per 100000 inhabitants)	**278.6**	189	75.2	77.6	67.6	197.9	224.4	**257.5[18]**	154.6	83.3[18]	61.3	88.0	..	172.9[17]
Hospital discharges for hypertensive diseases (per 100000)	**279.9**	52.9	27.8	58.8	111.9	169.2	54.8	**300.5[18]**	51.0	21.1[18]	182.8	56.3	..	44.6[17]
Hospital discharges for heart failure (per 100000 inhabitants)	**369.4**	278.8	129.9	203.7	98.7	366.9	136.7	**548.1[18]**	356.7	149.3[18]	152.2	266.4	..	228.7[17]
Hospital discharges for acute myocardial infarction (per 100000)	**258.6**	115.3	162.9	131.9	128.4	231	57.7	**275.7[18]**	177.5	164.7[18]	126.4	224.4	..	55.2[17]
Hospital discharges for angina pectoris (per 100000)	**376**	201.5	151.9	35.3	94.1	14.7	254.1	**262.8[18]**	92.2	54.5[18]	28.0	69.4	..	302.9[17]
Hospital discharges for cerebrovascular diseases (per 100000)	**497.2**	210.1	208.7	221.5	207.4	236.9	452.5	**542[18]**	259.9	217.4[18]	237.2	324.2	..	560.3[17]
Hospital discharges for atherosclerosis (per 100000 inhabitants)	**182.4**	86	12.7	36.0	66.0	42.5	25.2	**241.8[18]**	90.6	19.6[18]	45.1	105.1	..	51.9[17]
Hospital discharges for alcoholic liver disease (per 100000 inhabitants)	**42.9**	42.6	27.9	26.0	18.7	21.5	36.9	**45[18]**	26.4	31.2[18]	24.9	24.5	..	22.5[17]
Hospital discharges for malignant neoplasm of trachea, bronchus, and lung (per 100000 inhabitants)	**223.4**	69	82.3	70.5	72.5	46.2	188.8	**244.2[18]**	69.2	57.0[18]	75.2	112.8	..	310.5[17]
Average length of stay for patients with acute myocardial infarction (AMI), days	**11.1**	6.6	9.8	9.0	7.2	5.5	..	**9.8[18]**	5.2	6.2[18]	7.1	6.3	..	..
30-day mortality after admission to hospital for AMI (age-sex standardized rate per 100 patients aged 45+)	**11.0**	7.9	..	10.6	9.1	6.8	..	**8.3**	5.6[15]	6.6	6.5	..	4.9[18]	9.7[17]

Notes: [d] - different methodology or break in series; [e] - OECD estimate; [p] - provisional figure; [06] - 2006; [07] – 2007; [09] – 2009; [14] – 2014; [15] - 2015; [16] – 2016; [17] – 2017; [18] – 2018.

Supplementary table S2 (continued). Major characteristics of health system financing, resources, and performance and health risk factors in Germany (DEU), France (FRA), the United Kingdom (UK), Spain (ESP), Switzerland (SWI), the USA, and Japan (JPN). OECD estimates [17, 20] for 2005 and 2019 (or nearest year).

Indicator	2005							2019						
	DEU	FRA	UK	ESP	SWI	USA	JPN	DEU	FRA	UK	ESP	SWI	USA	JPN
**Health behavior and risk factors**														
% of females aged 15+ who are daily smokers (self-reported survey)	**18.8**	23.2[d]	23.0	21.5[06]	17.6[17]	14.9	11.3	**15.3[17]**	20.7	13.8	18.8[17]	16.8[17]	10.0	7.6
% of males aged 15+ who are daily smokers (self-reported survey)	**27.9**	32.0[d]	25.0	31.6[06]	23.4[07]	19.1	39.3	**22.3[17]**	27.5	17.7	25.6[17]	21.5[17]	11.7	27.1
Tabacco consumption in grams per capita (sales data)	**1978**	1297	1134[e]	..	2046	1807	2595	**1503**	841	420.6[e]	1473	1228	1004	1065
Cigarettes per smoker per day (survey self-reported data)	**14.7[09]**	13.8[06]	14.0	20.9[06]	11.7[07]	16.7	19.6	**15.2**	12.5[d]	9.1	12.3[17]	10.0[17]	13.7	15.5
Annual consumption of pure alcohol in liters, per person, aged 15 years old and over	**11.7**	12.9	11.4	11.9	10.1	8.5	8.5	**10.6**	11.4	9.7	10.7	9.3	8.9	7.1
Sugar supply (all forms of sugar and sweeteners) in kg per capita	**50.9**	41.7	37.6	29.9	58.9	70.4	28.7	**44.2[18]**	42.6[18]	38.0[18]	33.2[18]	48.6[18]	64.2[18]	26.0[18]
All vegetable supply in kg per capita per year	**86.2**	104.2	95.9	155.8	88	123	107.8	**89.8[18]**	99.1[18]	77.3[18]	124.8[18]	92.8[18]	112.9[18]	92.9[18]
All fruit supply in kg per capita per year	**87.1**	112.1	127	100.2	71.9	109.8	60.3	**78.1[18]**	82.9[18]	77.5[18]	94.6[18]	83.8[18]	90.2[18]	34.1[18]
% of females aged 15+ eating vegetables (excl. potatoes and juice) at least once per day (survey)	**53.8[09]**	..	..	47.8[06]	90.9[d,07]	91.6[07]	..	**42.5[14]**	63.3[14]	70.0[14]	48.4[17]	76.2[17]	92.5[18]	..
% of males aged 15+ eating vegetables (excl. potatoes and juice) at least once per day (survey)	**37.2[09]**	..	..	36.2[06]	82.9[d,07]	93.4[07]	..	**25.3[14]**	51.4[14]	60.3[14]	35.7[17]	59.1[17]	91.0[18]	..
% of females aged 15+ eating fruits at least once per day (survey)	**71.5[09]**	..	..	72.0[06]	89.1[d,07]	61.6[07]	..	**55.6[14]**	61.2[14]	67.9[14]	70.2[17]	67.9[17]	57.7[18]	..
% of males aged 15+ eating fruits at least once per day (survey)	**52.5[09]**	..	..	62.5[06]	77.9[d,07]	54.3[07]	..	**38.7[14]**	48.5[14]	56.9[14]	59.3[17]	51.3[17]	50.1[18]	..

Notes: [d] - different methodology or break in series; [e] - OECD estimate; [p] - provisional figure; [06] - 2006; [07] – 2007; [09] – 2009; [14] – 2014; [15] - 2015; [16] – 2016; [17] – 2017; [18] – 2018.
