# Supplementary material for: The underwhelming German life expectancy
Source: Eur J Epidemiol. 2023 Apr 25;38(8):839–50. doi: 10.1007/s10654-023-00995-5 (PMC10129301; doi:10.1007/s10654-023-00995-5)
Supplement: Supplementary file 2 — Supplementary Material 2 [file 10654_2023_995_MOESM2_ESM.pdf]

Article: The underwhelming German life expectancy. European Journal of Epidemiology.
Authors: Domantas Jasilionis, Alyson van Raalte, Sebastian Klüsener, Pavel Grigoriev.
The corresponding author: Domantas Jasilionis, Laboratory of Demographic Data, Max Planck Institute for Demographic Research, Rostock, Germany. E-mail: jasilionis@demogr.mpg.de

Fig. S1 Trends in life expectancy at birth in Germany (total), West Germany, East Germany, and other six selected high-income countries, 1955 (or earliest available year) - 2020.

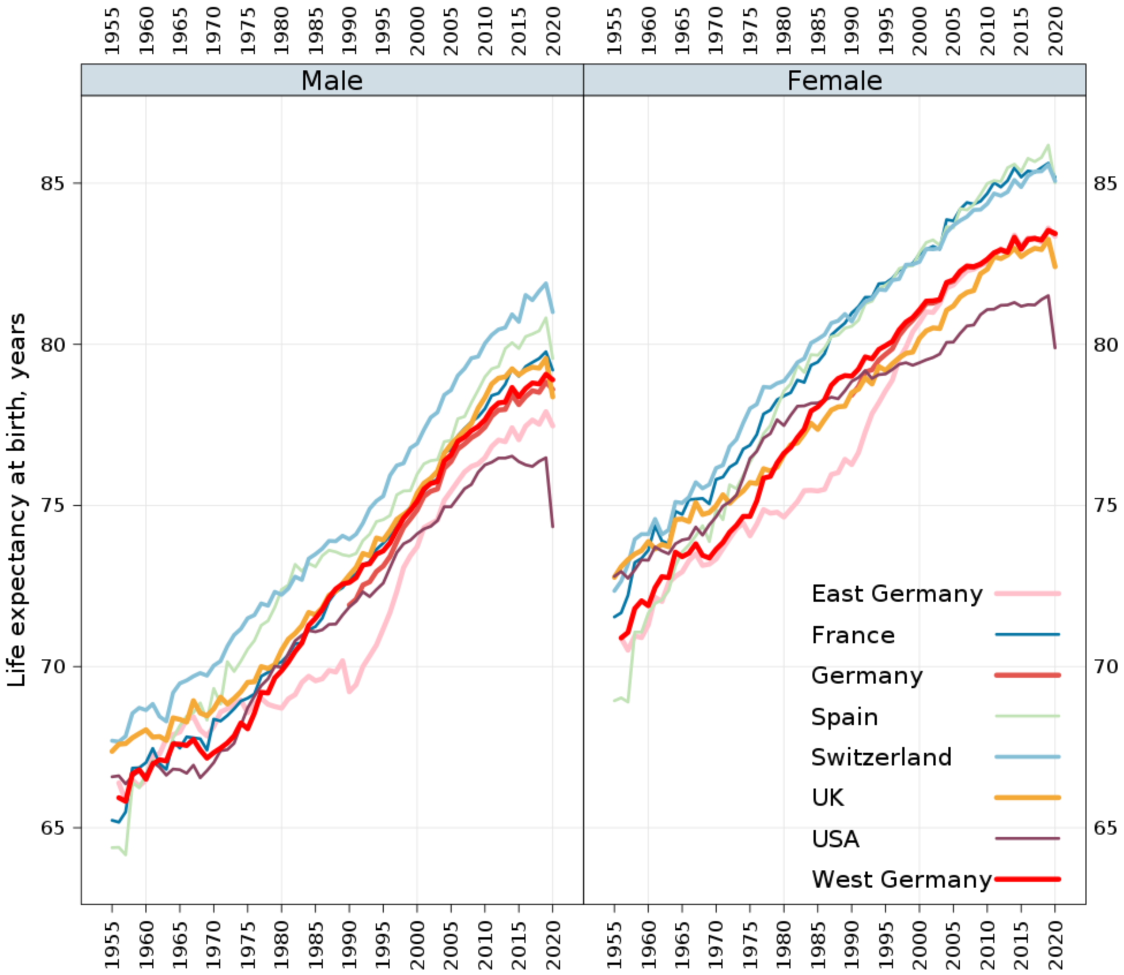

Data source: Human Mortality Database (HMD). University of California, Berkeley (USA) and Max Planck Institute for Demographic Research (Germany). Available at www.mortality.org (data downloaded on [5 January 2023]).

Fig. S2 Recent trends in age-standardized death rates for cardiovascular system diseases at ages 50-64 and 65+, 2001-2016.

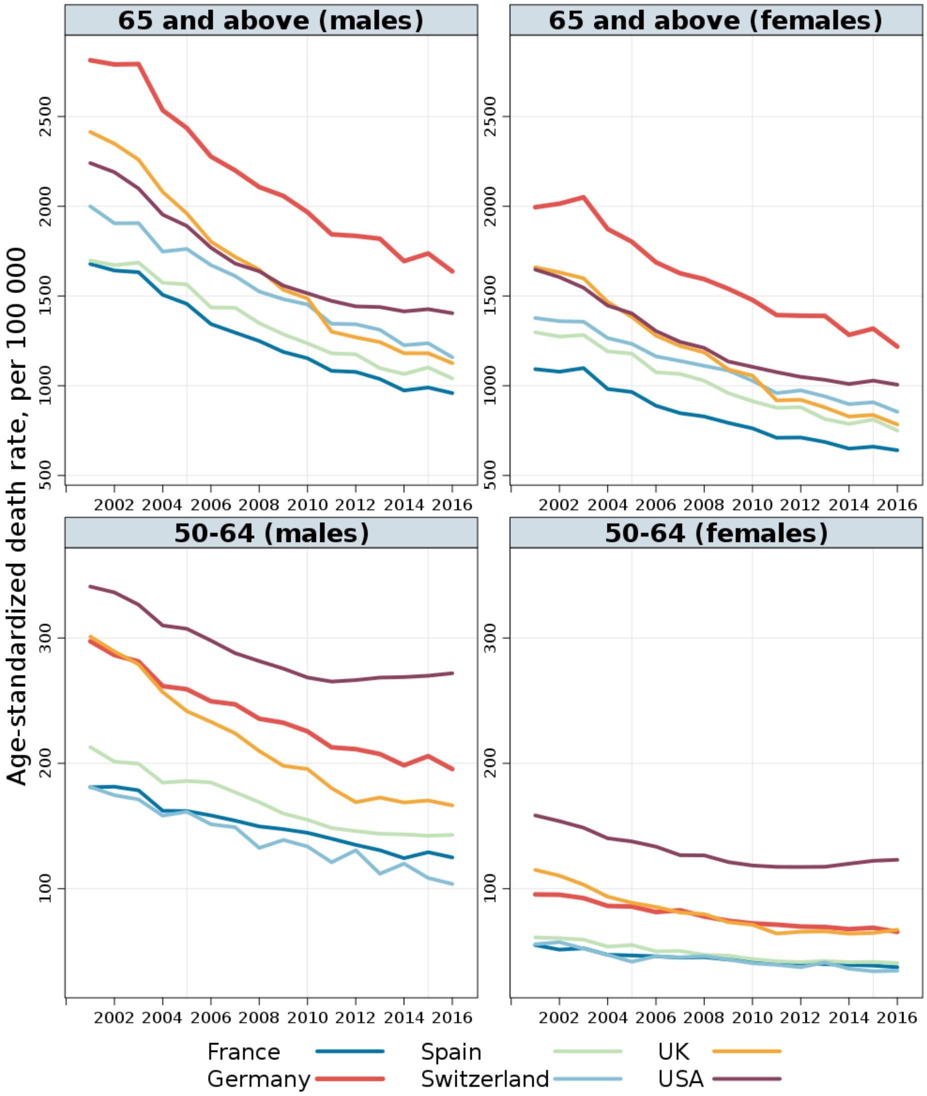

Data source: World Health Organization (WHO). WHO Mortality Database. Available at: http://www.who.int/data/data-collection-tools/who-mortality-database (data downloaded on [23 May 2022]).

Fig. S3 Age-specific contributions to the total difference in cohort temporary life expectancy between the exact ages 40 and 90 between Germany and each of the other selected high-income countries, cohorts born 1915-1930.

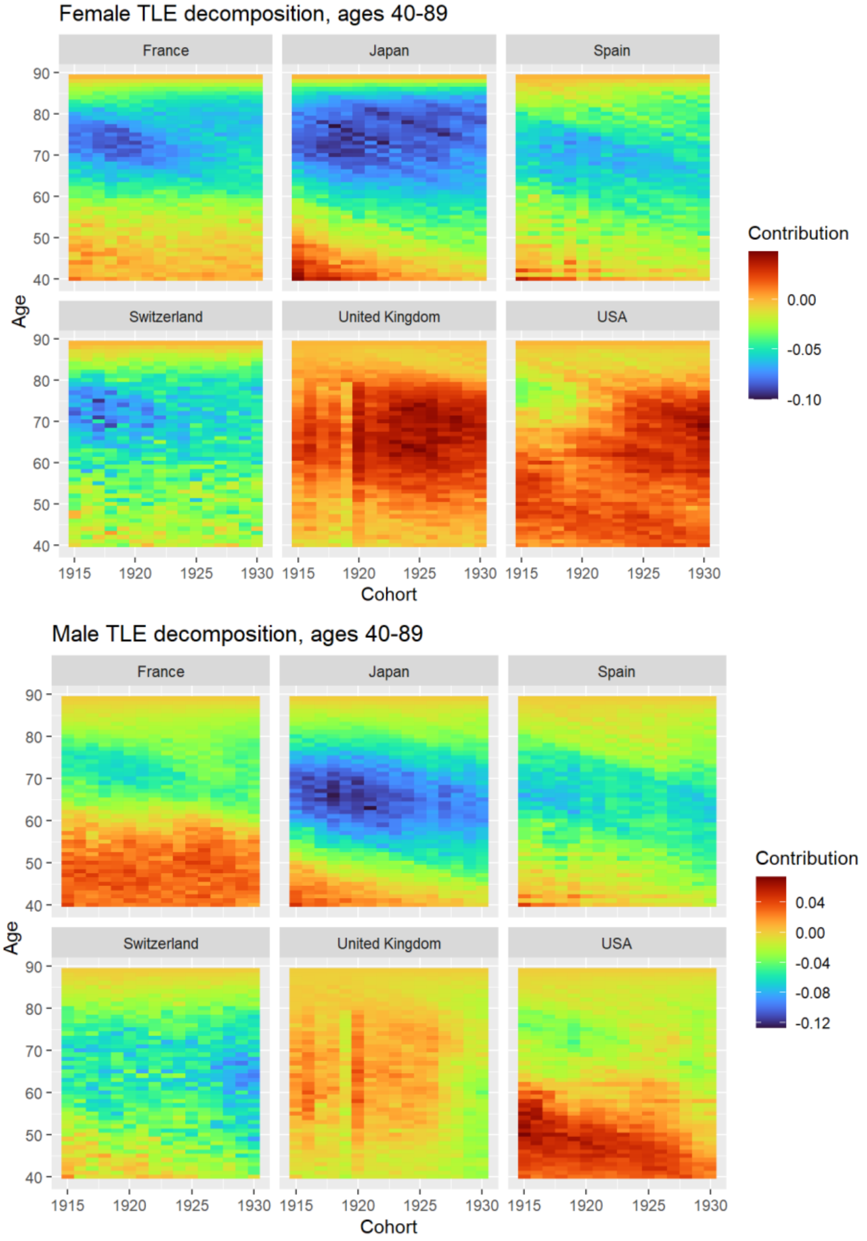

Data source: Human Mortality Database (HMD). University of California, Berkeley (USA) and Max Planck Institute for Demographic Research (Germany). Available at www.mortality.org (data downloaded on [5 January 2023]).
